# Prognostic capacity of the systemic inflammation response index for functional outcome in patients with aneurysmal subarachnoid hemorrhage

**DOI:** 10.3389/fneur.2023.1054315

**Published:** 2023-03-02

**Authors:** Yuyang Hou, Jingxin Fan, Huisheng Yuan, Hu Zheng, Hongkuan Yang, Hua Li, Rudong Chen, Jiasheng Yu

**Affiliations:** ^1^Department of Neurosurgery, Tongji Hospital, Tongji Medical College, Huazhong University of Science and Technology, Wuhan, Hubei, China; ^2^Department of Neurosurgery, Hubei Provincial Hospital of Integrated Traditional Chinese and Western Medicine, Wuhan, Hubei, China

**Keywords:** inflammation, functional outcome, systemic inflammation response index (SIRI), aneurysm, subarachnoid hemorrhage

## Abstract

**Objective:**

We aimed to investigate the relationship between systemic inflammatory response index (SIRI) and functional outcome after aneurysmal subarachnoid hemorrhage (aSAH).

**Methods:**

A retrospective cohort study was performed involving all consecutive aSAH patients admitted to our institution. The modified Rankin Scale (mRS) score was performed to determine the functional outcomes of all patients at 3 months after aSAH. Results were categorized as favorable (mRS score 0–2) and unfavorable (mRS score 3–6). Univariate and multivariate logistic regressive analyses were utilized to identify the prognostic significance of SIRI. To minimize the effects of confounding factors, patients were stratified according to the optimal cut-off value of SIRI with propensity score matching (PSM). Further subgroup analysis was conducted to verify the consistency of our findings and Pearson's correlation analysis was used to assess the relationship between SIRI and the severity of aSAH.

**Results:**

In this study, 350 patients were enrolled and 126 (36.0%) of them suffered unfavorable outcomes. The SIRI of 5.36 × 10^9^/L was identified as the optimal cut-off value. Two score-matched cohorts (*n* = 100 in each group) obtained from PSM with low SIRI and high SIRI were used for analysis. A significantly higher unfavorable functional outcome rate was observed in patients with high SIRI before and after PSM (*p* < 0.001 and 0.017, respectively). Multivariate logistic regression analysis demonstrated that SIRI value ≥ 5.36 × 10^9^/L was an independent risk factor for poor outcomes (OR 3.05 95% CI 1.37–6.78, *p* = 0.006) after adjusting for possible confounders. A identical result was discovered in the PSM cohort. In ROC analysis, the area under the curve (AUC) of SIRI was 0.774 which shown a better predictive value than other inflammatory markers observed in previous similar studies. Pearson's correlation analysis proved the positive association between SIRI and aSAH severity.

**Conclusions:**

Elevated SIRI at admission is associated with worse clinical status and poorer functional outcomes among patients with aSAH. SIRI is a useful inflammatory marker with prognostic value for functional outcomes after aSAH.

## Introduction

Aneurysmal subarachnoid hemorrhage (aSAH) is a devastating hemorrhagic stroke with a high rate of mortality and disability (1). The deficits in language, memory, and executive function of some survivors who have suffered bleeding prevent them from regaining independence (2, 3). To improve the overall quality of survival in aSAH patients, biomarkers used to predict the outcomes may contribute to recognizing high-risk patients and directing appropriate treatment options. Systemic inflammation has been implicated to play a significant role in cerebrovascular disease, even though the precise mechanisms have not been adequately disclosed (4–7). Alterations in peripheral blood cell composition were considered to be indicative of a variety of inflammatory responses. Various novel composite markers were calculated on the foundations of these peripheral blood parameters to be used in the study of various diseases. Several inflammatory markers such as platelet-neutrophil ratio, platelet-lymphocyte ratio, and combined score of fibrinogen and neutrophil-lymphocyte ratio have been identified in prior studies to correlate with clinical outcomes following aSAH (8–10).

Systemic inflammatory response index (SIRI), a composite inflammatory marker based on neutrophil, monocyte, and lymphocyte counts, was utilized widely in oncology studies (11–13). However, fewer studies have explored its clinical significance in patients with aSAH (14, 15). For this retrospective study, we attempted to investigate if SIRI measured with blood samples on admission was affiliated with functional outcomes in patients with aSAH and to estimate the predictive value.

## Materials and methods

### Patient population

A retrospective analysis of data from all consecutive patients treated for subarachnoid hemorrhage (SAH) due to aneurysm rupture from January 2018 to March 2022 at the Department of Neurosurgery, Tongji Hospital, Tongji Medical College, Huazhong University of Science and Technology was performed. The inclusion criteria were as follows: (1) spontaneous SAH was confirmed by Computerized Tomography (CT) or lumbar puncture; (2) specific diagnosis of intracranial aneurysm (IA) detected by Computerized Tomography Angiography (CTA), Digital Subtraction Angiography (DSA), or surgery; (3) Patient with aSAH treated with surgical clipping or endovascular coiling within 72 h; (4) age of patients > 18 years; (5) adequate laboratory parameters on admission could be collected in advance of treatment. Participants were excluded if there was a status as listed below: (1) patients with brain injury or other cerebrovascular diseases such as arteriovenous malformations, dural arteriovenous fistulas, the relationship between SAH and aneurysm cannot be determined; (2) surgical treatment was not performed within 72 h for the aSAH patient; (3) patient loss of follow-up within 3 months; (4) past medical history of kidney or liver dysfunction and autoimmune; (5) data of neutrophil, monocyte and lymphocyte counts at the time of admission were not available.

### Laboratory and clinical data

All blood samples were gathered within 24 h of the patient's admission to the hospital. Both demographic variables (age and gender) and comorbidities and complications consisting of hypertension, diabetes, hyperlipidemia, smoking, and drinking alcohol were acquired. Furthermore, we obtained detailed peripheral blood cell counts on admission including leukocytes, neutrophils, lymphocytes, monocytes and platelets. Based on CT imaging, radiological data including modified Fisher scale (mFisher) scale, acute hydrocephalus, aneurysm location, intracerebral hemorrhage (ICH) and intraventricular hemorrhage (IVH) were also gained on admission. The treatments for aneurysms were equally included. All patients were evaluated by qualified neurosurgeons on admission and 3 months after release from the hospital. Glasgow Coma Score (GCS), Hunt-Hess grade as well as World Federation of Neurosurgical Societies (WFNS) grade were used to assess the initial clinical status. The neurological prognosis was determined by the modified Rankin Scale (mRS) score gained from a semi-structured telephone interview. According to the results of mRS scores, patients were categorized into favorable outcome (mRS score of 0–2) and unfavorable outcome groups (mRS score of 3–6). SIRI was defined as follows: SIRI = neutrophil count x monocyte count/lymphocyte count.

### Statistical analysis

Continuous variables were shown as mean ± standard deviation (SD) or median (interquartile range, IQR) depending on their distribution. Independent sample *t*-test or Mann-Whitney U-test was performed based on the normality of the distribution of continuous variables. Categorical variables were expressed as frequency with percentage and analyzed by the Chi-square test or Fisher exact test, as appropriate.

The optimal cut-off value was identified through the use of the maximization of Youden's index calculated by receiver operating characteristic (ROC) analysis. Based on the results of the optimal cut-off value of SIRI, patients were divided into two groups, high SIRI (≥5.36 × 10^9^/L) and low SIRI (< 5.36 × 10^9^/L). A 1:1 propensity score matching (PSM) with caliper width set as 0.20 was performed to minimize confounding bias. The imbalances of clinically relevant parameters including presence of ICH and IVH, hydrocephalus, pneumonia, Hunt-Hess grade, WFNS grade, GCS score, and mFisher grade between the two groups have been adjusted.

Univariate and multivariate logistic regression analyses have been performed for further illustration of the association between functional outcomes and SIRI among aSAH patients. In the crude model, no variates were adjusted. In model 1, demographic variables (age and gender) were adjusted. Apart from demographic factors, in model 2 we adjusted for comorbidities and complications comprising hypertension, hyperlipidemia, diabetes mellitus, smoking, drinking, pneumonia and hydrocephalus. Model 3, built on the foundation of model 2, further adjusted for variables potentially involved in the severity of aSAH, such as GCS grade, Hunt-Hess grade, WFNS grade, mFisher grade, ICH and IVH. We selected factors as confounders follow the following principles: (1) a factor had a change in the effect estimate of more than 10%; (2) a factor was significantly associated with the outcomes of interest.

To gain a better understanding of the predictive value of SIRI, ROC curve analysis was used. The area under the curve (AUC) was calculated to estimate the performance of SIRI. To validate the consistency of the results, a subgroup analysis was carried out. Subgroup analysis was conducted *via* logistic regression models defined by age (< 65 vs. ≥65 years), gender, presence of ICH, presence of IVH, hydrocephalus, WFNS grade (< 4 vs. ≥4), mFisher grade (< 3 vs. ≥3), Hunt-Hess grade (< 4 vs. ≥4) and time from onset to blood draw (< 24 vs. ≥24). The Pearson correlation method was used to analyze the correlation between SIRI and WFNS, Hunt-Hess and mFisher grades. Statistical analyses were performed using SPSS 25.0 (SPSS Inc., Chicago, IL, USA) and R software version 3.6.3 (http://www.r-project.org). *P*-value < 0.05 was considered statistically significant.

## Results

### Patient characteristics

**Figure 1:**
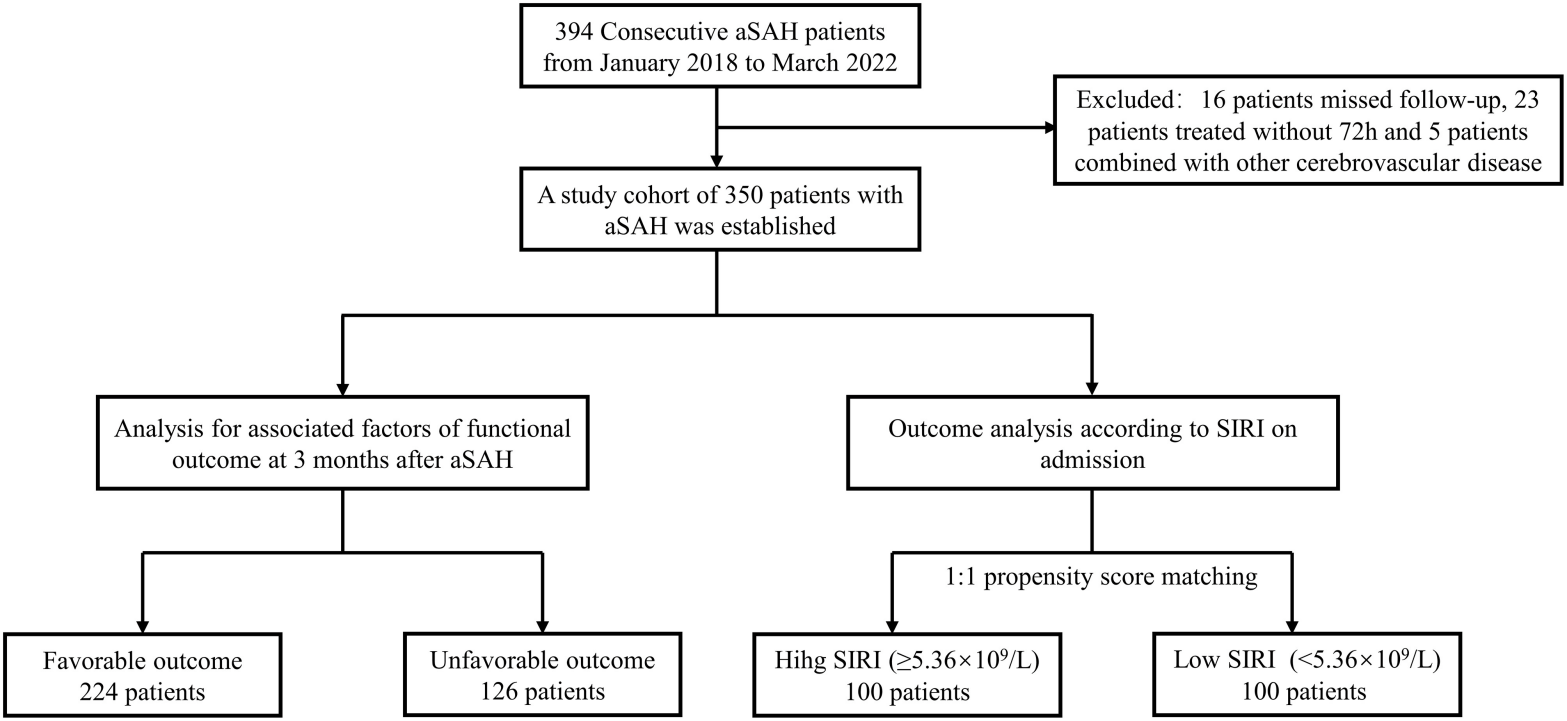
Study flowchart. aSAH, aneurysmal subarachnoid; hemorrhage; SIRI, systemic inflammatory response index.

A total of 394 consecutive patients with aSAH received treatment at our institution from January 2018 to March 2022 were recruited. As illustrated in Figure 1, 39 of them were excluded because of a variety of reasons. Finally, a cohort of 350 patients with 135 males (38.6%) and 215 females (61.4%) was acquired. According to the 3-month mRS score, 224 patients and 126 patients were assigned to favorable and unfavorable group, respectively. The baseline characteristics of the two groups were detailed in Table 1. Notable diversities were observed in the age, hypertension, diabetes mellitus, pneumonia and hydrocephalus between the two groups (*p* < 0.05). Moreover, the unfavorable group demonstrated worse initial clinical status with lower mean GCS scores [12 (IQR: 6–14) vs. 15 (IQR: 15–15), *p* < 0.001] and higher mean WFNS and Hunt-Hess grade [WFNS grade 4 (IQR: 2–5) vs. 1 (IQR: 1–1), *p* < 0.001; Hunt-Hess grade 3 (IQR: 3–4) vs. 1 (IQR: 1–1), *p* < 0.001]. The Occurrence of ICH and IVH among the unfavorable group was also remarkably higher than that of the favorable group (ICH: 46.8 vs. 11.6%, *p* < 0.001; IVH: 54.0 vs. 15.2%; *p* < 0.001). A distinct increase in mFisher grade was detected in the unfavorable group [3 (IQR: 2–4) vs. 1 (IQR: 1–2), *p* < 0.001]. There were no substantial variations in the location of the aneurysm or the treatment modality between the two groups. Among the laboratory parameters, the mean values of leukocyte count, neutrophil count, lymphocyte count, monocyte count and SIRI were revealed to be obviously higher in the unfavorable group (*p* < 0.05).

**Table 1:** Baseline characteristics of aSAH patients.

**Variables**	**Total (*n =* 350)**	**Favorable (*n =* 224)**	**Unfavorable (*n =* 126)**	***P*-value**
**Demographics**				
age, mean ± SD	55.6 ± 9.8	55.8 ± 9.3	57.1 ± 10.6	0.040
Female, *n* (%)	215 (61.4)	139 (62.1)	76 (60.1)	0.749
**Comorbidities**				
Hypertension, *n* (%)	153 (43.7)	89 (39.7)	64 (50.8)	0.045
Hyperlipidemia, *n* (%)	64 (18.3)	40 (17.9)	24 (19.0)	0.782
Diabetes mellitus, *n* (%)	30 (8.6)	11 (4.9)	19 (15.1)	0.001
Smoking, *n* (%)	90 (25.7)	58 (25.9)	32 (25.4)	0.919
Drinking, *n* (%)	101 (28.9)	61 (27.2)	40 (31.7)	0.371
**Clinical data**				
Admission Hunt-Hess grade, median (IQR)	2 (1–3)	1 (1–1)	3 (3–4)	< 0.001
Admission WFNS grade, median (IQR)	1 (1–3)	1 (1–1)	4 (2–5)	< 0.001
Admission GCS score, median (IQR)	15 (13–15)	15 (15–15)	12 (6–14)	< 0.001
Hydrocephalus, *n* (%)	44 (12.6)	13 (5.8)	31 (24.6)	< 0.001
Pneumonia, *n* (%)	80 (22.9)	13 (5.8)	67 (53.2)	< 0.001
Presence of ICH, *n* (%)	85 (24.3)	26 (11.6)	59 (46.8)	< 0.001
Presence of IVH, *n* (%)	102 (29.1)	34 (15.2)	68 (54.0)	< 0.001
mFisher grade, median (IQR)	1 (1–3)	1 (1–2)	3 (2–4)	< 0.001
**Aneurysm locations**				**0.541**
Middle cerebral artery, *n* (%)	81 (23.1)	53 (23.7)	28 (22.2)	
Anterior cerebral artery, *n* (%)	17 (4.9)	9 (4.0)	8 (6.3)	
Anterior communicating artery, *n* (%)	101 (28.9)	61 (27.2)	40 (31.7)	
Posterior communicating artery, *n* (%)	105 (30.0)	74 (33.0)	31 (24.6)	
Internal carotid artery, *n* (%)	22 (6.3)	10 (4.5)	12 (9.5)	
Posterior circulation, *n* (%)	17 (4.8)	12 (5.4)	5 (4.0)	
Others, *n* (%)	7 (2.0)	5 (2.2)	2 (1.6)	
**Treatment**				**0.617**
Coiling, *n* (%)	117 (33.4)	77 (34.4)	40 (31.7)	
Clipping, *n* (%)	233 (66.6)	147 (65.6)	86 (68.3)	
**Laboratory parameters on admission, median (IQR)**				
Glucose, mmol/L	7.0 (6.0–8.4)	6.6 (5.7–7.6)	8.1 (6.6–10.1)	< 0.001
Platelet count, × 10^9^/L	202.0 (161.5–247.5)	205.5 (170.8–245.2)	189.0 (149.0–247.5)	0.088
Hemoglobin, g/L	129.0 (118.0–140.0)	130.0 (119.8–139.0)	128.5 (117.2–140.0)	0.491
Red blood cells count, × 10^9^/L	4.3 (3.9–4.6)	4.3 (4.0–4.6)	4.3 (3.8–4.7)	0.921
Leukocytes count, × 10^9^/L	11.0 (8.2–13.7)	9.6 (7.6–12.0)	13.2 (10.8–17.2)	< 0.001
Neutrophil count, × 10^9^/L	9.2 (6.5–12.2)	8.0 (6.0–10.3)	11.7 (9.2–15.3)	< 0.001
Lymphocyte count, × 10^9^/L	0.9 (0.7–1.3)	1.1 (0.8–1.4)	0.8 (0.6–1.1)	< 0.001
Monocyte count, × 10^9^/L	0.5 (0.4–0.7)	0.5 (0.4–0.7)	0.6 (0.4–0.9)	< 0.001
NLR	9.7 (5.6–15.9)	7.2 (4.5–11.1)	15.7 (10.4–22.9)	< 0.001
PLR	206.1 (153.0–287.7)	190.8 (143.1–260.6)	225.8 (170.6–319.5)	< 0.001
SIRI, × 10^9^/L	4.8 (2.7–9.3)	3.6 (2.1–6.0)	8.6 (5.0–14.7)	< 0.001

aSAH, aneurysmal subarachnoid hemorrhage; SD, standard deviation; IQR, interquartile range; WFNS, World Federation of Neurosurgical Societies; GCS, Glasgow Coma Scale; ICH, intracerebral hemorrhage; IVH, intraventricular hemorrhage; mFisher, modified Fisher; NLR, neutrophil to lymphocyte ratio; PLR, platelet to lymphocyte ratio; SIRI, systemic inflammation response index.

### Association of SIRI with functional outcome

The optimal cut-off value of SIRI obtained with the maximization of Youden's index from the ROC analysis was 5.36 × 10^9^/L. Allowing for the effects of confounding factors and selection bias, the 1:1 PSM was developed to enhance the dependability of the results. The propensity scoring was performed as per IVH, mFisher grade, WFNS grade and GCS score with the caliper width set as 0.20. As shown in Table 2, both the high and low SIRI cohorts (100 individuals in each group) yielded after PSM shown a well balance in clinically relevant parameters and were available for further analysis (*p* < 0.05). There was a remarkable increase in the proportion of patients with poor prognosis at 3 months after aSAH which could be observed in aSAH patients with high SIRI both before and after matching (before PSM: 61.0 vs. 16.3%, *p* < 0.001; after PSM: 42.0vs. 26.0%, *p* = 0.017).

**Table 2:** The clinical characteristics and outcomes of aSAH patients before and after PSM.

**Characteristic**	**Before PSM**				**After PSM**			
	**All patients**	**Low SIRI**	**High SIRI**	***P*** **-value**	**All patients**	**Low SIRI**	**High SIRI**	***P*** **-value**
***N***	**350**	**196**	**154**		**200**	**100**	**100**	
Admission WFNS grade, median (IQR)	1 (1–3)	1 (1–1)	2 (1–4)	< 0.001	1 (1–2)	1 (1–2)	1 (1–2)	0.284
Admission GCS score, median (IQR)	15 (13–15)	15 (15–15)	13 (8–15)	< 0.001	15 (14–15)	15 (14–15)	15 (14–15)	0.440
mFisher grade median (IQR)	1 (1–3)	1 (1–2)	2 (1–3)	< 0.001	2 (1–3)	1 (1–3)	2 (1–3)	0.541
Admission Hunt-Hess grade, median (IQR)	2 (1–3)	1 (1–2)	3 (1–4)	< 0.001	2 (1–3)	2 (1–3)	2 (1–3)	0.833
Presence of ICH, *n* (%)	85 (24.3)	24 (12.2)	61 (39.6)	< 0.001	43 (21.5)	18 (18)	25 (25)	0.228
Presence of IVH, *n* (%)	102 (29.1)	35 (17.9)	67 (43.5)	< 0.001	68 (34.0)	33 (33)	35 (35)	0.765
Hydrocephalus, *n* (%)	44 (12.6)	17 (8.7)	27 (17.5)	0.013	26 (13.0)	13 (13)	13 (13)	1
**Functional outcome**				< 0.001				0.017
Favorable	224 (64.0)	164 (83.7)	60 (39)		132 (66.0)	74 (74)	58 (58)	
Unfavorable	126 (36.0)	32 (16.3)	94 (61)		68 (34.0)	26 (26)	42 (42)	

aSAH, aneurysmal subarachnoid hemorrhage; PSM, propensity score matching; SIRI, systemic inflammation response index; WFNS, World Federation of Neurosurgical Societies; IQR, interquartile range; GCS, Glasgow Coma Scale; mFisher, modified Fisher; ICH, intracerebral hemorrhage; IVH, intraventricular hemorrhage.

To independently analyze the effect of SIRI on the prognosis of aSAH, univariate and multivariable logistic regression analyses were conducted. And the results were displayed in Table 3. Crude model was a univariate logistic analysis without adjusting any parameters, SIRI was related to the unfavorable outcome after aSAH (OR 1.17, 95% CI 1.12–1.23, *p* < 0.001). In model 1, age and gender were modified; except for demographic variables, model 2 was adjusted for comorbidities and complications (hyperlipidemia, diabetes mellitus, smoking, drinking, pneumonia and hypertension), we further modified factors that might be related to the severity of disease (GCS grade, Hunt-Hess grade, WFNS grade, mFisher grade, ICH and IVH) in model 3. The results of multivariable logistic regression analyses of the three models indicated that high SIRI (≥5.36 × 10^9^/L) was an independent risk factor of poorer prognosis in aSAH patients (Model 1: OR 9.46, 95% CI: 5.59–15.99, *p* < 0.001; Model 2: OR 8.75, 95% CI: 4.65–16.45, *p* < 0.001; Model 3: OR 3.05, 95% CI: 1.37–6.78, *p* = 0.006). The similar results were also observed in the PSM cohort (Table 3).

**Table 3:** Multivariate logistic regression analysis for functional outcome in aSAH patients before and after PSM.

**Characteristic**	**Crude**		**Model 1**		**Model 2**		**Model 3**	
	**OR (95% CI)**	***P*** **-value**	**OR (95% CI)**	***P*** **-value**	**OR (95% CI)**	***P*** **-value**	**OR (95% CI)**	***P*** **-value**
**Before PSM**								
SIRI, × 10^9^/L	1.17 (1.12–1.23)	< 0.001	1.19 (1.13–1.25)	< 0.001	1.17 (1.11–1.23)	< 0.001	1.09 (1.02–1.17)	0.009
Low SIRI (< 5.36 × 10^9^/L)	1 (Ref)		1 (Ref)		1 (Ref)		1 (Ref)	
High SIRI (≥5.36 × 10^9^/L)	8.03 (4.88–13.22)	< 0.001	9.46 (5.59–15.99)	< 0.001	8.75 (4.65–16.45)	< 0.001	3.05 (1.37–6.78)	0.006
**After PSM**								
SIRI, × 10^9^/L	1.06 (1–1.11)	0.039	1.06 (1.01–1.13)	0.028	1.08 (1.01–1.15)	0.025	1.08 (1–1.17)	0.046
Low SIRI (< 5.36 × 10^9^/L)	1 (Ref)		1 (Ref)		1 (Ref)		1 (Ref)	
High SIRI (≥5.36 × 10^9^/L)	2.06 (1.13–3.75)	0.018	2.41 (1.27–4.57)	0.007	2.88 (1.34–6.23)	0.007	3.27 (1.25–8.56)	0.016

Model 1: Adjusted for age, gender.

Model 2: Model 1 plus comorbidities and complications (pneumonia, hydrocephalus, hypertension, hyperlipidemia, diabetes mellitus, smoking and drinking).

Model 3: Model 2 plus variables related to disease severity (GCS grade, Hunt-Hess grade, WFNS grade, mFisher grade, ICH and IVH).

aSAH, aneurysmal subarachnoid hemorrhage; PSM, propensity score matching; OR, odds ratio; CI, confidence interval; SIRI, systemic inflammation response index; GCS, Glasgow Coma Scale; WFNS, World Federation of Neurosurgical Societies; mFisher, modified Fisher; ICH, intracerebral hemorrhage; IVH, intraventricular hemorrhage.

### Prognostic performance of SIRI in predicting functional outcome for aSAH

**Figure 2:**
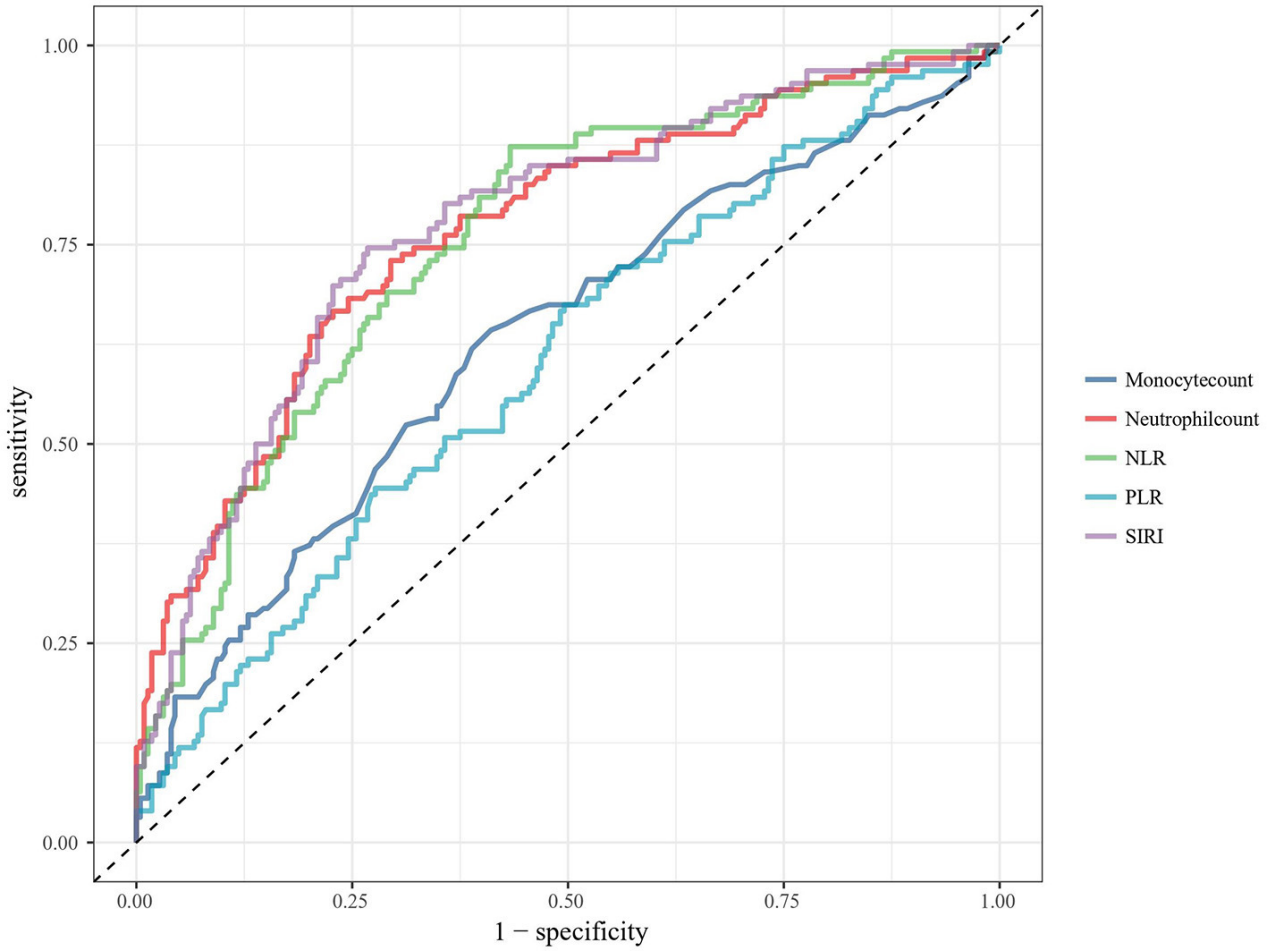
ROC analysis of the association between biomarkers and functional outcome at 3 months after aSAH. ROC, receiver operating characteristic; aSAH, aneurysmal subarachnoid hemorrhage; NLR, neutrophil to lymphocyte ratio; PLR, platelet to lymphocyte ratio; SIRI, systemic inflammation response index.

To better understand the predictive performance of SIRI, ROC analyses were performed to compare the precision, accuracy, sensitivity, specificity, positive and negative predictive values of SIRI and other inflammatory markers identified in similar previous studies. The curve of ROC analysis was shown in Figure 2. The overall performance of these biomarkers was illustrated in Table 4. Admission SIRI level of 5.36 × 10^9^/L was observed to have the best cut-off value. The AUC of SIRI was 0.774 (95% CI: 0.723–0.826) as this was the largest among neutrophil and monocyte count, neutrophil to lymphocyte ratio (NLR), platelet to lymphocyte ratio (PLR) and SIRI. The sensitivity and specificity of SIRI were 0.746 (95% CI: 0.659–0.817) and 0.732 (95% CI: 0.668–0.788). The positive predictive value and negative predictive value of SIRI were 0.610 (95% CI: 0.528–0.687) and 0.837 (95% CI: 0.776–0.884), respectively. Furthermore, the positive likelihood ratio and negative likelihood ratio were 2.785 (95% CI: 2.192–3.538) and 0.347 (95% CI: 0.256–0.469), respectively. Admission SIRI had better accuracy than other inflammatory factors for predicting functional outcomes of aSAH patients at 3 months.

**Table 4:** Overall performance of the biomarkers according to the ROC analysis.

	**NLR**	**PLR**	**SIRI**	**Neutrophil count**	**Monocyte count**
Cut-off value	8.12	191.00	5.36 × 10^9^/L	10.49 × 10^9^/L	0.525 × 10^9^/L
AUC (95% CI)	0.758 (0.706–0.810)	0.601 (0.540–0.663)	0.774 (0.723–0.826)	0.767 (0.714–0.819)	0.631 (0.569–0.693)
FP	97	111	60	51	92
FN	16	41	32	42	45
Sensitivity (95% CI)	0.873 (0.799–0.923)	0.675 (0.585–0.754)	0.746 (0.659–0.817)	0.667 (0.576–0.747)	0.643 (0.552–0.725)
Specificity (95% CI)	0.567 (0.499–0.632)	0.504 (0.437–0.572)	0.732 (0.668–0.788)	0.772 (0.710–0.824)	0.589 (0.522–0.654)
PPV (95% CI)	0.531 (0.461–0.601)	0.434 (0.364–0.506)	0.610 (0.528–0.687)	0.622 (0.534–0.703)	0.468 (0.393–0.545)
NPV (95% CI)	0.888 (0.822–0.933)	0.734 (0.655–0.800)	0.837 (0.776–0.884)	0.805 (0.744–0.854)	0.746 (0.674–0.807)
LR. positive (95% CI)	2.016 (1.711–2.375)	1.361 (1.138–1.629)	2.785 (2.192–3.538)	2.928 (2.233–3.839)	1.565 (1.277–1.919)
LR. negative (95% CI)	0.234 (0.141–0.356)	0.645 (0.497–0.837)	0.347 (0.256–0.469)	0.432 (0.336–0.554)	0.606 (0.477–0.771)
Accuracy	0.677	0.566	0.737	0.734	0.609
Precision	0.531	0.434	0.610	0.622	0.468

NLR, neutrophil to lymphocyte ratio; PLR, platelet to lymphocyte ratio; SIRI, systemic inflammation response index; AUC, area.

under the curve; CI, confidence interval; FP, false positive; FN, false negative; PPV, positive predictive value; NPV, negative predictive value; LR, likelihood ratio.

### Subgroup analysis

**Figure 3:**
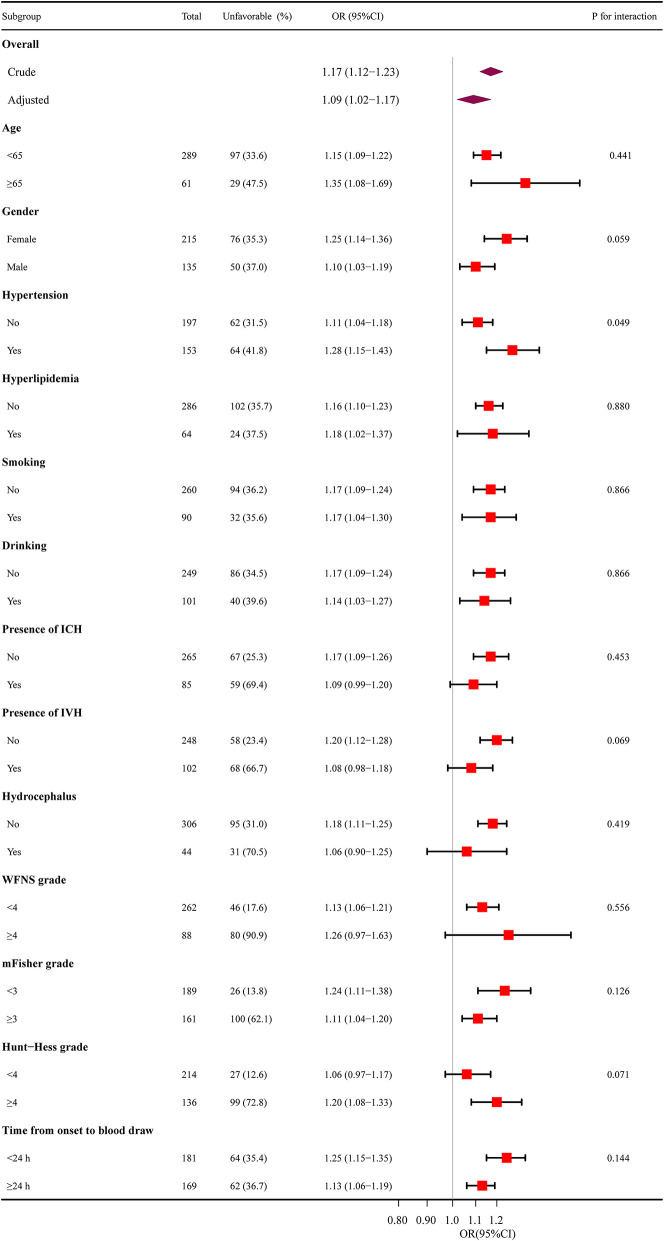
The relationship between SIRI and functional outcomes in subgroup analysis. SIRI, systemic inflammation response index; ICH, intracerebral hemorrhage; IVH, intraventricular hemorrhage; WFNS, World Federation of Neurosurgical Societies; mFisher, modified Fisher; OR, odds ratio; CI, confidence interval.

Subgroup analysis was performed to evaluate the interaction of age (< 65 and ≥65 years), gender, hypertension, hyperlipidemia, smoking, drinking, IVH, ICH, hydrocephalus, WFNS grade (< 4 and ≥4), mFisher grade (< 3 and ≥3), Hunt-Hess grade (< 4 and ≥4) and time from onset to blood draw (< 24 and ≥24) on SIRI (Figure 3). No statistically significant interactions between the SIRI and functional outcome in aSAH patients were observed except for hypertension (*P* for interaction = 0.049).

### Association between SIRI and severity of aSAH

**Figure 4:**
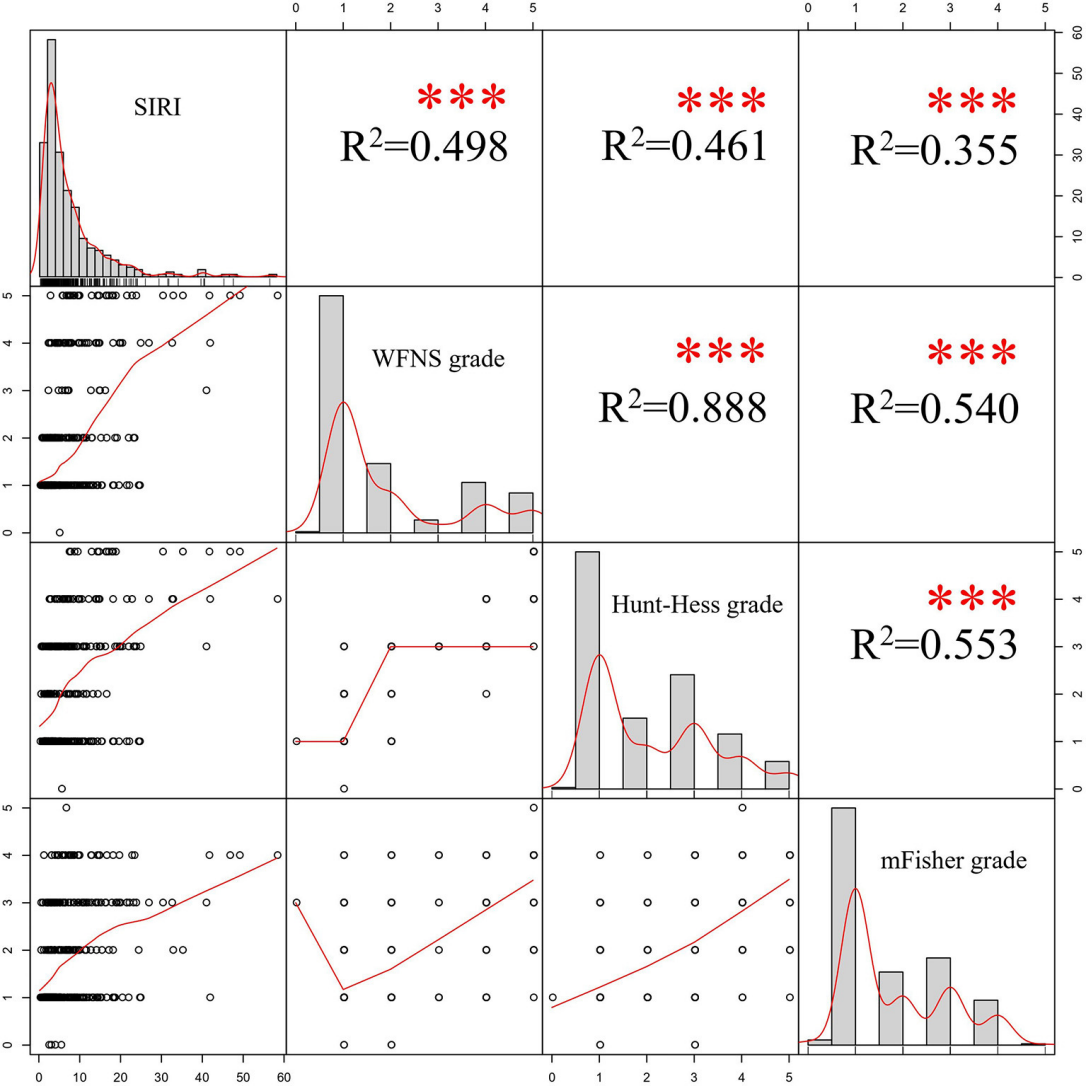
Results of Pearson's correlation analyses. SIRI, systemic inflammation response index; WFNS, World Federation of Neurosurgical Societies; mFisher, modified Fisher.

The results of the Pearson correlation analyses were shown in Figure 4. Positive correlations were able to be observed among SIRI and WFNS, Hunt-Hess and mFisher grades, the correlation coefficient was 0.498, 0.461 and 0.355, respectively (*p* < 0.001).

## Discussion

In recent years, the impacts of systemic inflammation in aSAH have captured the interest of researchers and various novel inflammatory markers such as have been used to predict the prognosis of aSAH (16–18). Nevertheless, tests of these biomarkers are not routinely used in clinical applications owing to the cost, technical expertise and equipment setup. Therefore, exploring easily accessible inflammatory markers is essential. SIRI emerged as a marker of systemic inflammation based on neutrophil, monocyte, and lymphocyte counts. Moreover, it could be obtained with no extra techniques or costs. All these laboratory parameters used to calculate SIRI are simple, rapid, non-invasive, inexpensive and widely applicable.

As is commonly known, neutrophils are the major players in the inflammatory response and they can contribute to inflammation *via* various inflammatory factors or mechanisms. For instance, both free oxygen radicals and protein hydrolases are products of neutrophils which perform crucial roles in the pathogenesis of cerebral blood barrier damage (19). Besides, previous researchers have considered the production of neutrophil extracellular traps (NETs) as a process that helps neutrophils catch and destroy germs. A growing amount of evidence indicates that NETs may also occur in non-infectious, sterile inflammation (20). Hanhai Zeng et al. discovered that the formation of NETs exacerbated neuroinflammation after SAH by facilitating the activation of microglia (21). Monocytes may also affect the prognosis of subarachnoid hemorrhage by promoting neuroinflammation through different mechanisms. Santiago et al. identified peripheral monocytes count on admission as a risk factor for cerebral infarction and unfavorable functional outcome after aSAH (22). Aaron et al. found monocyte count shown a gradually increase after SAH and reached its peak on day 6–8, and it was associated with delayed cerebral ischemia (DCI) and adverse clinical outcomes (23). Matthew et al. revealed that elevated level of soluble growth stimulation expressed gene 2 (sST2) after aSAH may be relevant to increased expression of CD16^+^ monocytes, thus compromising the inflammatory response causing neurological deterioration (24). As an essential portion of inflammation, lymphocytes represent the resolution of inflammation (25). Individual paraments might be inadequate to illustrate the severity of inflammation. Therefore, composite biomarkers of inflammation by combining different leukocyte subtypes are extensively accepted. SIRI could reflect both anti-inflammatory response mediated by lymphocytes and proinflammatory response mediated by neutrophils and monocytes.

SIRI as a composite biomarker has been commonly discussed in oncology researches (26–28). However, rare attention has been devoted to this inflammatory marker in the studies of cerebrovascular disease. Zhang et al. discovered a positive correlation between SIRI and stroke severity (29). Zhang et al. revealed that increased SIRI was related to poorer outcomes (Glasgow outcome scale 1–3) after aSAH (15), but it was limited by the small sample size. In current study, we investigated the prognostic effects of SIRI on aSAH systematically and comprehensively using laboratory parameters collected at admission. The imbalances of different clinically relevant parameters between high and low SIRI group have been adjusted by using PSM to minimize the impact of confounding factors on the results. Regardless of before and after PSM, the incidence of unfavorable outcome was significantly higher in the high SIRI group (Table 2). Variation in the duration between aneurysm rupture and blood sampling may produce unknown impact on the results of SIRI. Subgroup analysis was conducted to assess the interaction of interval time on SIRI. A total of 181 (51.7%) patients had their blood specimens drawn within 24 h and 64 (35.4%) of them shown poor outcome. The interval time between aSAH attack and blood sampling was longer than 24 h in the remaining 169 (48.3%) patients and 62(36.7%) of them shown poor outcome. No statistically significant interaction between the SIRI and interval time before sampling was observed (P for interaction = 0.144). Furthermore, increased SIRI at admission was considered to be associated with unfavorable outcome in aSAH patients at 3 months depending on the results of univariate and multivariate logistic regression analyses. The subgroup analysis suggests that our findings are reliable. To demonstrate the prognostic predictive capacity of SIRI and inflammatory markers identified in previous studies on functional outcomes after aSAH, ROC analysis was conducted. In comparison with other inflammatory markers such as neutrophil count, monocyte count, NLR and PLR, SIRI was detected to be a more reliable indicator of prognosis in patients with aSAH. It was generally recognized that the higher the WFNS and Hunt-Hess scores, the worse the clinical status. In addition, higher mFisher grades were commonly regarded as a symbol of a larger amount of bleeding in subarachnoid. In our cohort, SIRI was positively correlated with WFNS, Hunt-Hess and mFisher grades (*p* < 0.001), so we could draw a conclusion that SIRI and severity of aSAH were positively associated. After PSM to adjust for confounders, it was noted that SIRI predicted prognosis for patients with aSAH independently of WFNS, Hunt-Hess and mFisher grades.

## Limitations

There are several limitations of current study should be noted, such as the nature of its retrospective single-center study. Prospective multicenter studies are necessary to resolve this concern. The interval time between aneurysm rupture and admission to our institution varies extensively among patients, which may cause unknown bias in results of laboratory parameters. Frequent testing of these laboratory parameters and calculating averages might probably reduce the bias due to different time intervals.

## Conclusion

To summarize, SIRI was associated with the severity of aSAH and poorer outcome. Moreover, SIRI shown a better performance in predicting aSAH prognosis when compared to neutrophil and monocyte count, NLR and PLR. With consideration of cost and convenience, SIRI may provide useful information for the clinical management of patients with aSAH. Further studies are required to confirm these results and to clarify the mechanisms involved.

## Data availability statement

The raw data supporting the conclusions of this article will be made available by the authors, without undue reservation.

## Author contributions

JY contributed to the conception and design of the study, had full access to all the data in the study, and take responsibility for the integrity of the data and the accuracy of the data analysis. YH and RC wrote the statistical analysis plan, cleaned, and analyzed the data. JF, HYu, HZ, HL, and HYa contributed to the analysis and interpretation of the data. All authors participated in manuscript writing, revision, and approval for final submission.
